# Complete Genome Sequence of *Winogradskyella* sp. Strain PG-2, a Proteorhodopsin-Containing Marine Flavobacterium

**DOI:** 10.1128/genomeA.00490-14

**Published:** 2014-05-29

**Authors:** Yohei Kumagai, Susumu Yoshizawa, Kenshiro Oshima, Masahira Hattori, Wataru Iwasaki, Kazuhiro Kogure

**Affiliations:** a Atmosphere and Ocean Research Institute, the University of Tokyo, Kashiwa, Chiba, Japan; b CREST, Japan Science and Technology Agency, Kawaguchi, Japan; c Department of Computational Biology, Graduate School of Frontier Sciences, the University of Tokyo, Kashiwa, Chiba, Japan

## Abstract

*Winogradskyella* sp. strain PG-2 is a marine flavobacterium isolated from surface seawater. This organism contains proteorhodopsin, which can convert light energy into available forms of biochemical energy. Here, we present its complete genome sequence and annotation, which provide further insights into the life strategy of proteorhodopsin-mediated phototrophy in the ocean.

## GENOME ANNOUNCEMENT

Proteorhodopsin (PR), a seven-transmembrane domain protein containing retinal as a chromophore, functions as a light-driven proton pump (1). Culture-independent surveys revealed that PR genes are widely distributed among marine prokaryotes (2, 3). To acquire further insights into the ecological and physiological roles of PR, more studies based on genomic information and physiological experiments are required. Although some genomic and physiological information for PR-containing bacteria is now available (4–9), more detailed physiological studies such as quantitative analysis of proton-pumping activities are still lacking for many isolates. Here, we present the complete genome sequence of a marine flavobacterium strain, PG-2. The light-driven proton-pumping activity of PR in this organism was directly measured using native bacterial cells (10).

Strain PG-2 was isolated from seawater samples collected in the Sagami Bay (35°00′N, 139°20′E; depth, 100 m). The 16S rRNA sequence analysis revealed that strain PG-2 belongs to genus *Winogradskyella* (11).

The genome sequence of strain PG-2 was determined using 454 pyrosequencing by single-end (SE) and paired-end (PE) (8-kb-span library) data. We generated 135,373 reads by SE and 327,726 reads by PE sequencing, representing 46.5-fold coverage of the genome. Genome assembly was performed using Newbler v. 2.7 (454 Life Science), which generated five scaffolds. Gap closing and resequencing of low-quality regions in the assembled data were performed by PCR and primer walking using Sanger sequencing. Overall accuracy of the finished sequence was estimated to have an error rate of less than one per 10,000 bases (quality value of ≥40). The complete genome of PG-2 consisted of a 3,811,479-bp chromosome with an average G+C content of 32.2%. Open reading frame (ORF) calling and annotation were performed using the RAST server (11). The genome contained 3,527 protein-coding sequences, 2 rRNAs, and 54 transfer RNAs.

The genome of strain PG-2 contained a full-length PR gene. A *blh* gene encoding a 15,15′-β-carotene dioxygenase, whose gene product is predicted to produce two molecules of retinal from β-carotene, was located adjacent to the PR gene. Similar to other PR-containing *Bacteroidetes* (12), the reading frames of the PR and *blh* genes were oriented in opposite directions. In addition, all genes required for β-carotene biosynthesis, including farnesyl pyrophosphate and isopentenyl pyrophosphate (geranylgeranyl pyrophosphate synthase [*crtE*], phytoene synthase [*crtB*], phytoene desaturase [*crtI*], and lycopene cyclase [*crtY*] [13–15]) genes, were located in the genome of strain PG-2. With more sequence data and physiological studies of PR-containing bacteria, the genome of strain PG-2 should provide further insight into the physiological details of PR-mediated light utilization by surface-dwelling oceanic microbial species.

### Nucleotide sequence accession number.

This whole-genome shotgun project has been deposited in DDBJ/ENA/GenBank under the accession no. AP014583. The version described in this paper is the first version.
